# In Vitro Screening of Butcher’s Broom (*Ruscus aculeatus* L.) Cladodes and Aerial Parts Extracts Against Fish Pathogen Isolates

**DOI:** 10.3390/vetsci13090945

**Published:** 2026-09-11

**Authors:** İbrahim Demirkale, Hasibullah Bikzad, Miray Etyemez Büyükdeveci, Faik Sertel Secer

**Affiliations:** 1 Department of Aquaculture, Faculty of Fisheries, Cukurova University, Adana 01330, Türkiye; idemirkale@cu.edu.tr (İ.D.); bikzadhasibullah91@gmail.com (H.B.); metyemez@cu.edu.tr (M.E.B.); 2 Department of Fisheries and Aquaculture, Faculty of Agriculture, Ankara University, Ankara 06110, Türkiye; 3 Aquaterra Ltd., Ankara University Technopolis, Ankara 06120, Türkiye

**Keywords:** aquaculture, antimicrobial resistance, disc diffusion, inhibition zone, GC–MS, phytotherapy

## Abstract

The growing need for sustainable alternatives to antibiotics in aquaculture has increased interest in plant-derived antimicrobial compounds. This study characterized ethanol and methanol extracts from the cladodes and aerial parts of butcher’s broom (*Ruscus aculeatus* L.) by GC–MS and screened, as a preliminary in vitro assessment, their antibacterial activity against *Aeromonas hydrophila*, *Lactococcus garvieae*, *Vibrio anguillarum*, *Edwardsiella tarda*, and *Yersinia ruckeri*. The extracts showed distinct chemical profiles. Phytol showed the highest relative chromatographic abundance in the ethanol cladode extract (25.16% of the total ion chromatogram peak area), whereas n-hexadecanoic acid dominated the ethanol aerial parts extract (42.08%). In the methanol cladode extract, the most abundant peak was a co-eluting group (2,2-dimethylbutane/1,2-dimethylaziridine/oxalic acid isobutyl pentyl ester; 22.21%), followed by methyl 9,12,15-octadecatrienoate (14.64%) and phytol (7.86%), while the methanol aerial parts extract was characterized by a 4H-pyran-4-one derivative (16.41%). Antibacterial activity varied with extract, pathogen, and dose, with maximum inhibition zones of 25.0 ± 1.0 mm against *A. hydrophila*, 24.0 ± 1.0 mm against *Y. ruckeri*, and 23.7 ± 0.6 mm against *V. anguillarum*. The *E. tarda* isolate was the least susceptible. These preliminary findings provide the first evidence of antibacterial activity of *R. aculeatus* extracts against fish pathogens and support further investigation of this species as a potential source of antimicrobial phytochemicals for aquaculture.

## Simple Summary

Bacterial diseases are a major challenge in fish farming, and the frequent use of antibiotics to control these infections can contribute to the development of antimicrobial resistance. Plants containing naturally active compounds may provide new options for developing safer approaches to disease control. In this study, extracts prepared from the cladodes and aerial parts of butcher’s broom (*Ruscus aculeatus*) using ethanol or methanol were screened for their chemical composition and their ability to inhibit five important disease-causing bacteria of farmed fish. Chemical analysis showed that the extracts differed considerably in their major natural compounds. Their antibacterial effects also varied depending on the plant part, extraction solvent, bacterial species, and amount tested. Several extracts produced marked inhibition of bacterial growth, with the strongest effects observed against three of the tested fish pathogens, whereas one species was considerably less susceptible. These findings suggest that butcher’s broom warrants further investigation as a potential source of natural antimicrobial agents against bacterial diseases in farmed fish, with the long-term potential to contribute to reduced reliance on antibiotics in aquaculture.

## 1. Introduction

Aquaculture has emerged as one of the fastest-growing food-production sectors worldwide and is expected to make an increasingly important contribution to meeting the global demand for high-quality animal protein [1]. Nevertheless, the continued intensification of aquaculture production has created conditions that facilitate the emergence and transmission of infectious diseases. Bacterial infections, in particular, remain a major constraint on sustainable fish production because they can cause substantial mortality, impair growth and production performance, and result in considerable economic losses. Among the bacterial pathogens frequently associated with freshwater and marine aquaculture are the Gram-negative species *Aeromonas hydrophila*, *Vibrio anguillarum*, *Yersinia ruckeri*, and *Edwardsiella tarda*, together with the Gram-positive pathogen *Lactococcus garvieae* [2].

Antibiotics have traditionally represented one of the principal approaches to the control of bacterial diseases in cultured fish. However, their extensive or inappropriate use has contributed to the emergence and dissemination of antimicrobial-resistant bacteria and resistance determinants within aquatic environments [3]. This growing concern, together with the potential accumulation of antimicrobial residues and their effects on non-target organisms, has intensified the search for safer and more sustainable approaches to disease management in aquaculture [4]. In this context, naturally derived antimicrobial compounds have attracted particular interest because many are biodegradable and may exert lower environmental impacts than conventional synthetic chemotherapeutics [5]. Plant-derived phytochemicals are therefore increasingly being investigated as potential alternatives or complementary agents for controlling bacterial infections in aquatic animals [6].

Medicinal plants have long been recognized as a rich source of structurally diverse secondary metabolites, and plant-derived compounds such as terpenoids, alkaloids and flavonoids constitute the predominant part of the phytochemicals reported to possess antimicrobial activity against bacteria, fungi, viruses and protozoa [7]. This broad spectrum of biological activity has stimulated increasing interest in phytotherapeutic approaches in veterinary medicine, including aquaculture [8,9]. Experimental studies have already demonstrated that extracts from several medicinal plants can inhibit important bacterial fish pathogens. For example, ethanolic plant extracts have shown antibacterial activity against *Y. ruckeri*, *L. garvieae*, and *V. anguillarum* in disc-diffusion and microdilution assays [10]. Similarly, screening of 36 plant species from Türkiye revealed marked differences in susceptibility among fish pathogens, with *V. anguillarum* being among the most sensitive organisms tested [11]. Collectively, these findings support the continued exploration of medicinal plants as sources of candidate antimicrobial compounds for aquaculture applications [12].

Butcher’s broom, *Ruscus aculeatus* L., is a perennial evergreen subshrub belonging to the family Asparagaceae, subfamily Nolinoideae (formerly Ruscaceae), within the order Asparagales. A distinctive botanical feature of the species is that photosynthesis is performed predominantly by flattened, leaf-like stem structures known as cladodes rather than by true leaves [13,14]. The species is naturally distributed throughout much of the Mediterranean Basin and western Europe, where it is commonly associated with dry, shaded woodland and scrub habitats and exhibits considerable tolerance to the combined effects of shade and drought [13,15].

The pharmacological properties of *R. aculeatus* are largely associated with its rich secondary-metabolite profile. Particular attention has been given to steroidal saponins, especially ruscogenin- and neoruscogenin-related compounds, which occur predominantly in the rhizome but are also present in aerial parts [16,17]. In addition to steroidal saponins, the species contains phenolic compounds, flavonoids, sterols, triterpenes, coumarins, and other secondary metabolites that may contribute to its biological activity [18]. Saponins, in particular, are known to exhibit a wide range of biological effects, including antioxidant, hypolipidemic, and antimicrobial activities [19], while chemically related steroidal saponins have also been characterized in other members of the genus, including *Ruscus hyrcanus* [20].

A growing body of evidence supports the pharmacological potential of *R. aculeatus*. Extracts of the species have demonstrated antioxidant, anti-urease, and anticholinesterase activities [21], as well as antinociceptive and anti-inflammatory effects [22]. Antimicrobial activity has also been reported, with certain isolated compounds demonstrating inhibitory effects that are comparable to, or in some cases exceed, those of standard reference drugs [23]. Hydroethanolic and aqueous extracts of *R. aculeatus* have been shown to inhibit several clinically relevant microorganisms [18]. Likewise, ethyl acetate and chloroform extracts of the related species *Ruscus hypophyllum* displayed activity against both susceptible and multidrug-resistant bacterial strains, with reported minimum inhibitory concentrations (MIC) ranging from 0.125 to 0.5 mg/mL [24]. More recently, secondary metabolites obtained from the branches and cladodes of *R. aculeatus* have been evaluated against bacterial and fungal clinical isolates, with the observed activity largely confined to *Bacillus subtilis* ATCC 6633 [25], while methanolic extracts of plants collected in southern Türkiye have also shown measurable antimicrobial activity against *Staphylococcus aureus* and *Candida albicans* [26]. These observations further support the presence of biologically active antimicrobial constituents within the species.

Despite these findings, research on the antimicrobial properties of *R. aculeatus* has thus far focused almost exclusively on microorganisms of human clinical relevance [18,23,24,25]. To the best of our knowledge, its antibacterial activity against major bacterial pathogens of fish has not yet been investigated. Furthermore, phytochemical characterization of *R. aculeatus* has predominantly relied on HPLC-, LC–MS-, and NMR-based analytical approaches, whereas GC–MS profiling of its extracts remains comparatively limited [27]. Evaluating extracts obtained using solvents of differing polarity and from different plant fractions may therefore provide additional insight into both their chemical composition and their antibacterial potential.

Accordingly, the present study aimed to characterize the chemical composition of ethanol and methanol extracts prepared from the cladodes and aerial parts of *R. aculeatus* using GC–MS and to evaluate, as a preliminary screening, their in vitro antibacterial activity against five economically important bacterial fish pathogens. By integrating chemical profiling with antibacterial screening, this study seeks to provide an initial assessment of *R. aculeatus* as a potential source of naturally derived antimicrobial compounds for future applications in aquaculture health management.

## 2. Materials and Methods

### 2.1. Plant Material and Extract Preparation

**Figure 1:**
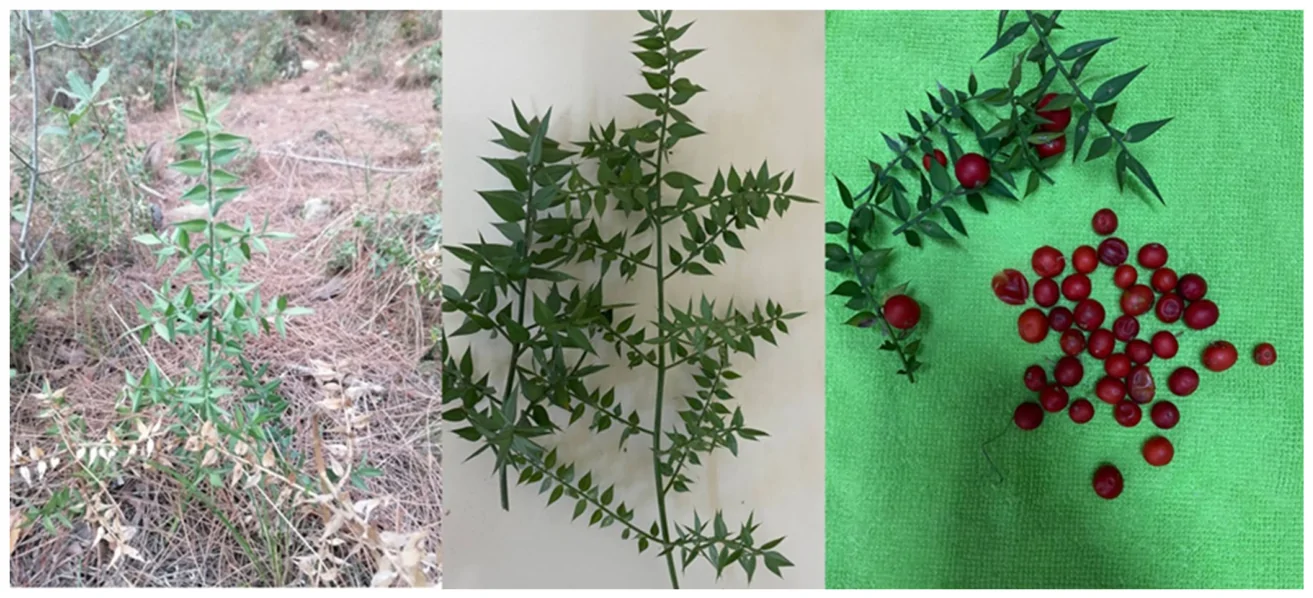
Photograph of the *R. aculeatus* plant taken at the Zorkun plateau (Osmaniye, Türkiye).

Plant material was collected along the lower slopes of Zorkun Plateau Road (Osmaniye, Türkiye), where *R. aculeatus* species occurs naturally at altitudes up to approximately 500 m (Figure 1). The plants were transported to the Fish Diseases Laboratory of the Faculty of Fisheries, Cukurova University, Türkiye, washed under running water and air-dried at room temperature.

A portion of the dried plants was separated into cladodes, whereas the remaining material, consisting of cladodes, stems, and fruits (excluding rhizome and roots), was processed as aerial parts. Ten grams of cladodes and aerial parts material were weighed out and placed into glass bottles with screw caps, to which 100 mL of ethanol (96%) and 100 mL of methanol (≥99.9%) were added (1:10, *w*/*v*), and the mixture was subjected to maceration; as a result of this process, four extraction groups were obtained: ethanol-extracted cladodes (EC), ethanol-extracted aerial parts (EA), methanol-extracted cladodes (MC) and methanol-extracted aerial parts (MA).

The bottles were kept in the dark at room temperature (22 ± 2 °C) for 7 days. The resulting mixtures were then filtered through Whatman No.4 filter paper (Whatman International Ltd., Maidstone, UK), and the filtrate was concentrated under reduced pressure using a rotary evaporator (Büchi Laboratory Equipment, Postfach, Switzerland). The solvent was removed to dryness and 100 mg of the dry residue of each extract was weighed and dissolved in 10 mL of the corresponding extraction solvent (96% ethanol or ≥99.9% methanol), giving a 10 mg/mL stock solution for each of the four extracts. Disc loadings of 100, 250 and 500 µg of dry extract per disc were obtained by applying 10, 25 and 50 µL of these stock solutions, respectively. The stock solutions were transferred to 10-mL amber glass vials and stored at +4 °C until analysis. Two millilitres of each stock solution were reserved for GC–MS characterization, and 3 mL were used for the disc diffusion assay.

### 2.2. Chemical Characterization by GC–MS

The extracts were transported under cold-chain conditions to the Central Research Laboratory of Cukurova University (Adana, Türkiye). Chemical constituents were determined using a gas chromatograph coupled to a mass spectrometer (7890B GC and 7010B MS; Agilent Technologies, Santa Clara, CA, USA) equipped with a DB-Wax capillary column (60 m × 0.25 mm i.d. × 0.25 µm film thickness; J&W Scientific, Folsom, CA, USA). One microliter of each sample, filtered through a 0.45 µm membrane filter, was injected into the capillary column at an injector temperature of 250 °C with a split ratio of 1:20. Helium (99.999% purity) was used as the carrier gas at a flow rate of 2 mL/min, operated in constant flow mode. The oven temperature was held at 40 °C for 4 min, increased to 90 °C at 3 °C/min, then to 130 °C at 4 °C/min and held for 4 min, and finally raised to 240 °C at 5 °C/min and held for 8 min. The ion source, quadrupole and MS transfer line temperatures were maintained at 240 °C, 150 °C and 280 °C, respectively. Mass spectra were acquired in electron impact mode at an electron energy of 70 eV over a mass range of 30–600 *m*/*z* in full-scan (MS1) mode; multiple reaction monitoring was not employed.

Constituents were tentatively identified by comparing their mass spectra with those of the NIST14 mass spectral library (NIST/EPA/NIH, version 2014). No internal standard, external calibration or response-factor correction was applied, and no absolute quantification was performed. The proportion of each constituent was calculated by area normalization of the total ion chromatogram, that is, the integrated peak area of each compound was divided by the summed area of all integrated peaks and expressed as a percentage. These values therefore represent relative chromatographic abundance rather than absolute concentrations.

### 2.3. Bacterial Strains and Antibacterial Assay

Five bacterial fish pathogens (*A. hydrophila* SY-AH2, *L. garvieae* SY-LG1, *V. anguillarum* SY-L24, *E. tarda* SY-ED1 and *Y. ruckeri* E42) previously isolated from diseased fish during field studies were used (Table 1). These strains were obtained from culture collection of the Faculty of Marine Sciences and Technology, Çanakkale Onsekiz Mart University, Türkiye. Species identity was confirmed by 16S rRNA gene sequencing of the isolates; the corresponding GenBank accession numbers are given in Table 1. The phenotypic and biochemical characteristics of the five isolates were determined in this study by conventional tests (Gram staining, cell morphology, motility, oxidase, catalase, oxidation/fermentation of glucose, indole production, H2S production, ornithine and arginine decarboxylase, mannitol and sucrose fermentation, and growth in the absence of NaCl) and were interpreted according to Buller [28]; the results are given in Table 2. Stock cultures were plated on tryptic soy agar and subsequently grown in tryptic soy broth for 24 h in a shaking incubator. The cultures were then centrifuged at 5000 rpm for 10 min, washed three times with sterile 0.9% NaCl, and each bacterial inoculum was adjusted to a 0.5 McFarland using a densitometer (DEN-1, BioSan, Warren, MI, USA), and the resulting densities were verified by viable counts for each of the five isolates (mean 1.2 × 10^8^ CFU/mL; range 1.1–1.3 × 10^8^ CFU/mL).

The antibacterial activity of the extracts was determined by the Kirby–Bauer disc diffusion method [30]. Sterile blank paper discs (Bioanalyse^®^ Dublin, Ireland; 6 mm diameter) were loaded with 100, 250 or 500 µg of dry extract per disc, applied as 10, 25 or 50 µL of the corresponding 10 mg/mL stock solution, in a laminar flow cabinet, allowed to dry completely, and stored at +4 °C in sterile Petri dishes until use. Negative-control discs impregnated with equal volumes of the corresponding extraction solvents (96% ethanol or ≥99.9% methanol), dried under the same conditions, and otherwise processed identically were included in each assay; no inhibition zones developed around these solvent-control discs against any of the five isolates, indicating that, after drying, residual solvent had no adverse effect on bacterial growth. Each bacterial suspension was spread onto Mueller–Hinton agar plates and allowed to dry, after which the extract-impregnated discs were placed on the agar surface at regular intervals. For comparison, a panel of ten commercial antibiotic discs (ampicillin 10 µg, imipenem 10 µg, enrofloxacin 5 µg, levofloxacin 5 µg, tetracycline 30 µg, oxytetracycline 30 µg, chloramphenicol 30 µg, streptomycin 10 µg, sulfamethoxazole-trimethoprim 25 µg and trimethoprim 5 µg (Oxoid, Basingstoke, Hampshire, UK)) were tested against the same isolates under identical conditions. The plates were incubated at 24 °C, and inhibition zone diameters were measured with a digital caliper after 48 h. For the halophilic *V. anguillarum* isolate (SY-L24), the assay was performed on Mueller–Hinton agar supplemented with NaCl (1.5% *w*/*v*) for its salinity requirement, and the extract-impregnated discs were placed on this salt-adjusted medium; the antibiotic discs and the corresponding solvent-control discs were likewise tested on the salt-supplemented plates and produced no inhibition zones. All five isolates produced confluent and homogeneous lawns under the assay conditions (24 °C, 48 h), allowing unambiguous zone measurement. Incubation at 24 °C was chosen to match the growth optima of the fish pathogens tested; because these conditions differ from those under which clinical interpretive breakpoints for the antibiotic discs were established, zone diameters were compared quantitatively and were not classified as susceptible or resistant. All assays, for both extracts and antibiotics, were performed in triplicate, with each replicate consisting of an independently grown bacterial culture plated on a separate agar plate.

### 2.4. Statistical Analysis

Inhibition zone diameters were recorded for all 60 extract–dose–isolate combinations of the design (4 extracts × 3 disc loadings × 5 isolates, *n* = 3). A combination was classified as inactive when no zone was detected in any replicate; because the disc itself was 6 mm in diameter, these observations lie at the detection floor of the assay and were entered into the analyses as measurements at that floor (zone diameter = 6 mm) rather than treated as missing values. The primary analysis was therefore performed on the complete 4 × 3 design of each isolate; because cells at the floor have zero within-cell variance, it was evaluated both by two-way ANOVA (extract x dose) and by the assumption-free Kruskal–Wallis test across the 12 extract–dose combinations (Table A5), and the Tukey comparisons shown in Table A1 were derived from this model. For comparison with the presentation customary for screening data, the same models were also fitted to the active combinations only; those results are reported in Table A3 and are conditional on activity, the extract and dose levels entering each model being specified in this study. Normality of the residuals was checked with the Shapiro–Wilk test and homogeneity of variances with the Brown–Forsythe (median-centred Levene) test. For each isolate with more than one active extract, inhibition zone diameters of the active combinations were analyzed by two-way analysis of variance (ANOVA; extract × dose, type II sums of squares) followed by Tukey’s HSD test for pairwise comparisons; because the Shapiro–Wilk test indicated mild departures from normality for *A. hydrophila* (*p* = 0.040) and *V. anguillarum* (*p* = 0.007), the results were additionally confirmed with the non-parametric Kruskal–Wallis test, applied to the active extract–dose combinations treated as a single factor, which yielded the same conclusions (Table A3). For the *E. tarda* isolate, only one extract–dose combination was active, and no inferential statistics were applied. The EC extract was likewise active against the A. hydrophila isolate at a single dose level only (500 µg per disc), so neither a regression nor a two-dose comparison was possible for this combination. Dose-dependence was additionally quantified by regressing zone diameter on log2-transformed dose for each extract–isolate combination with at least three active dose levels; combinations with only two active dose levels were summarized descriptively, because two points determine a linear slope mathematically and provide no information on the shape of the dose–response relationship. Each regression was therefore based on three dose levels (nine observations), as reported in Table A4; for combinations with only two active dose levels, the mean difference in zone diameter between the two doses is reported instead. Extract and antibiotic zone diameters (both *n* = 3) were compared by Welch’s *t*-tests at the highest disc loading (500 µg of dry extract per disc), with the Holm correction applied across all extract–antibiotic comparisons within each isolate; given the differences in diffusion behaviour, disc loading and formulation between crude extracts and purified antibiotics, these comparisons describe relative performance under the assay conditions rather than equivalence of potency. The level of significance was set at *p* < 0.05. Statistical analyses were performed with IBM SPSS Statistics (version 30; IBM Corp., Armonk, NY, USA); the Holm correction for the extract–antibiotic comparisons was applied to the SPSS-derived *p* values in a spreadsheet.

## 3. Results

### 3.1. Chemical Characterization of the Extracts

**Figure 2:**
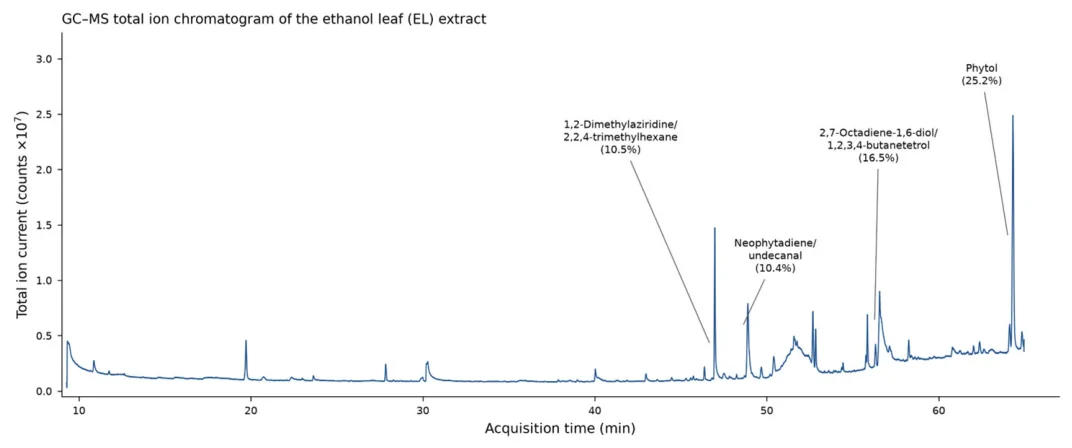
GC–MS total ion chromatogram of the EC extract of *R. aculeatus*. Major constituents (peak area ≥ 5%; Table 3) are annotated at their retention times with their relative peak areas.

**Figure 3:**
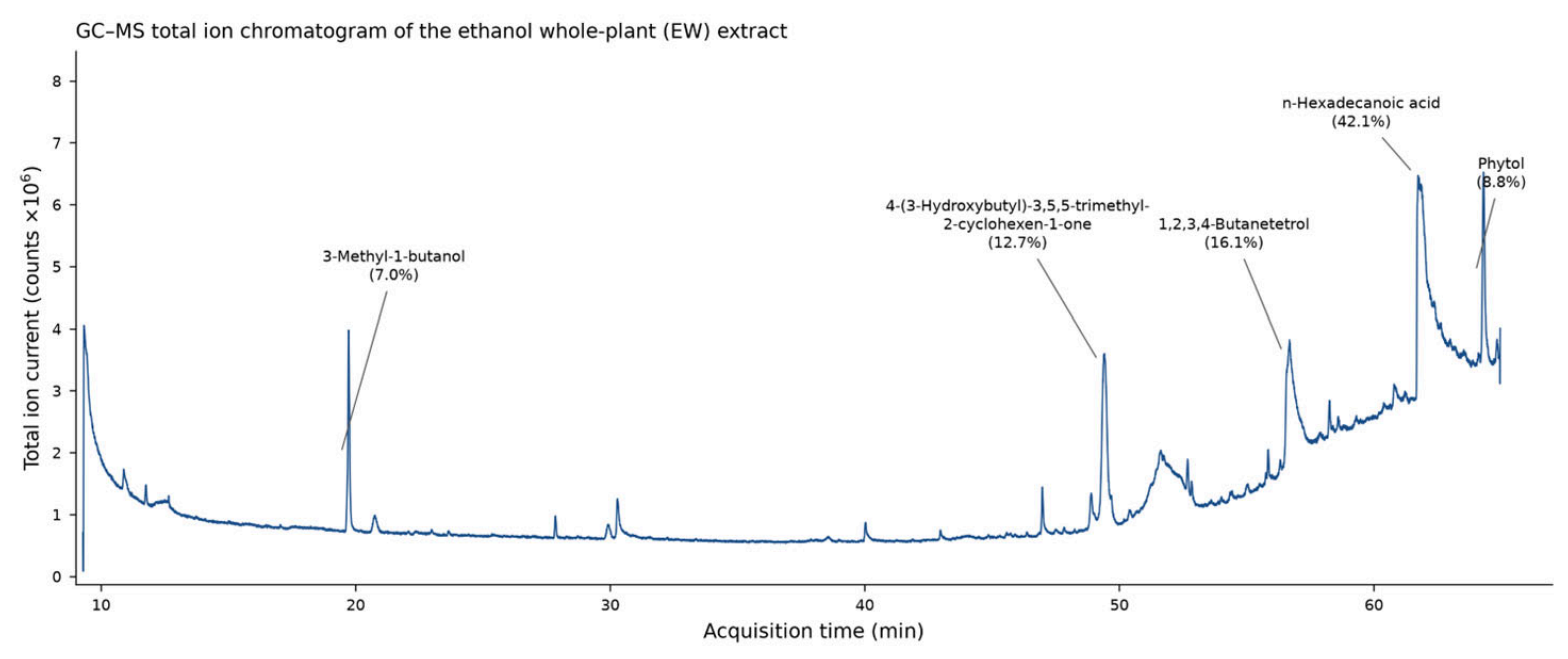
GC–MS total ion chromatogram of the EA extract of *R. aculeatus*. Major constituents (peak area ≥ 5%; Table 4) are annotated at their retention times with their relative peak areas.

**Figure 4:**
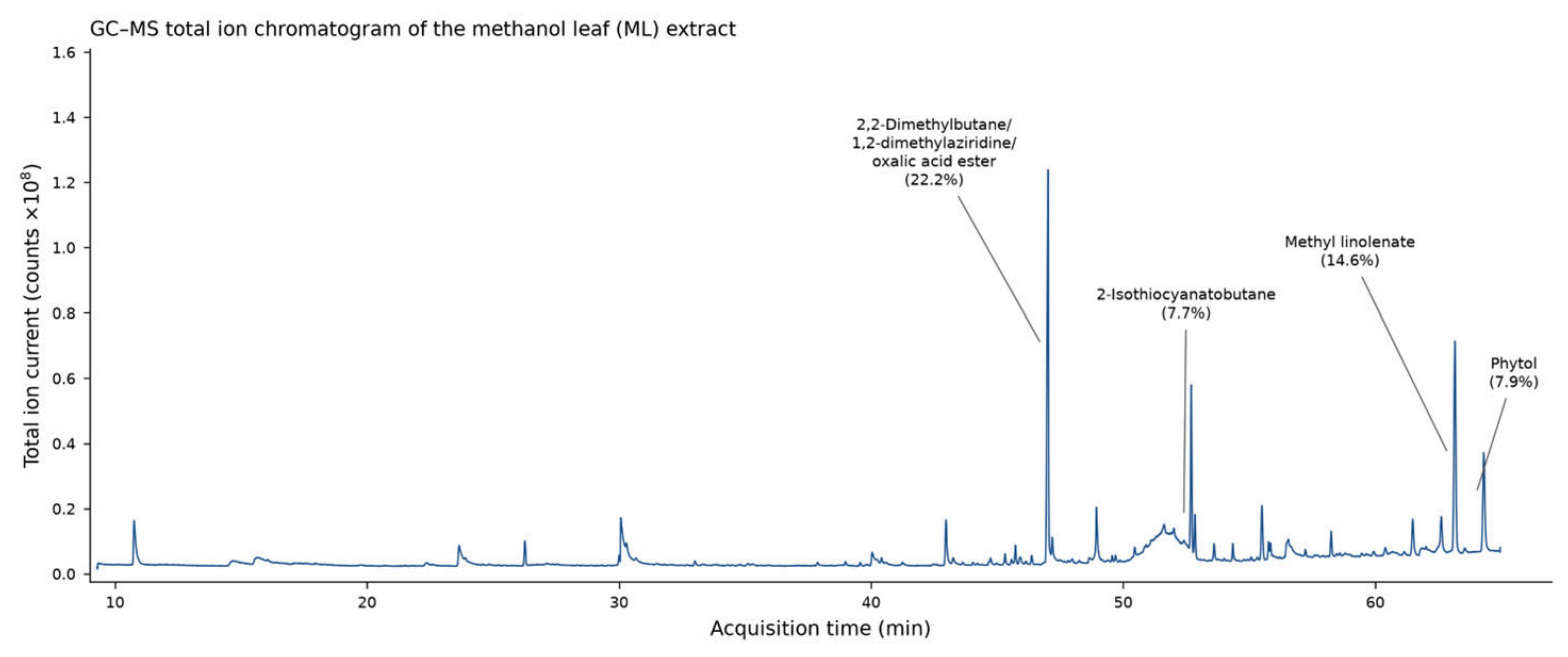
GC–MS total ion chromatogram of the MC extract of *R. aculeatus*. Major constituents (peak area ≥ 5%; Table 5) are annotated at their retention times with their relative peak areas.

**Figure 5:**
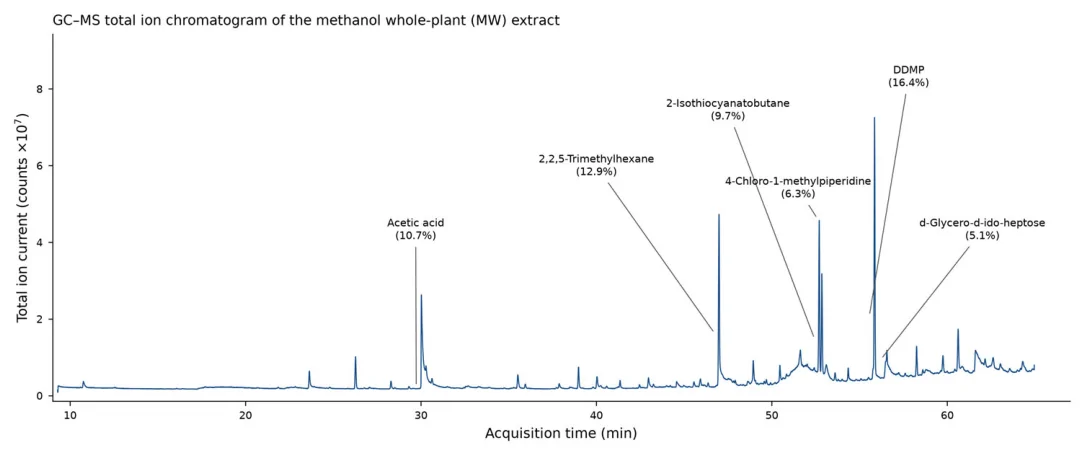
GC–MS total ion chromatogram of the MA extract of *R. aculeatus*. Major constituents (peak area ≥ 5%; Table 6) are annotated at their retention times with their relative peak areas.

The chemical composition of the four extracts, as tentatively determined by GC–MS, varied considerably with both the solvent and the plant part used. The major constituents (peak area ≥ 5%) of each extract are presented in Table 3, Table 4, Table 5 and Table 6 and Figure 2, Figure 3, Figure 4 and Figure 5. All values are expressed as the relative chromatographic abundance of each compound, obtained by area normalization of the total ion chromatogram.

A total of 32 compounds were tentatively identified in the EC extract, of which four exceeded 5% of the total peak area and together accounted for 62.6% of the chromatogram (Table 3, Figure 2). The diterpene alcohol phytol was the single most abundant constituent (25.16%), followed by the co-eluting pairs 2,7-octadiene-1,6-diol, 2,6-dimethyl/1,2,3,4-butanetetrol (16.52%), 1,2-dimethylaziridine/2,2,4-trimethylhexane (10.51%) and neophytadiene/undecanal (10.36%). The remaining 28 minor constituents each represented less than 5% of the total peak area (cumulatively 37.5%).

The EA extract showed the least complex profile, with 17 compounds detected, five of which exceeded the 5% threshold and jointly represented 86.5% of the total peak area (Table 4, Figure 3). n-Hexadecanoic acid (palmitic acid) clearly dominated the profile (42.08%), followed by 1,2,3,4-butanetetrol (16.05%), the megastigmane-type ketone 4-(3-hydroxybutyl)-3,5,5-trimethyl-2-cyclohexen-1-one (12.66%), phytol (8.77%) and 3-methyl-1-butanol (6.98%). The predominance of a saturated fatty acid distinguishes this extract from the cladode extracts, in which terpenoid constituents prevailed.

In the MC extract, 33 compounds were detected, four of which exceeded 5% of the total peak area (52.4% cumulatively) (Table 5, Figure 4). The most abundant peak corresponded to the co-eluting group 2,2-dimethylbutane/1,2-dimethylaziridine/oxalic acid isobutyl pentyl ester (22.21%), followed by methyl (Z,Z,Z)-9,12,15-octadecatrienoate (methyl linolenate; 14.64%), phytol (7.86%) and 2-isothiocyanatobutane (7.65%).

The MA extract was the most complex, with 38 detected compounds, six of which exceeded the 5% threshold and together accounted for 61.2% of the total peak area (Table 6, Figure 5). The pyranone derivative 2,3-dihydro-3,5-dihydroxy-6-methyl-4H-pyran-4-one (DDMP; 16.41%) was the principal constituent, followed by 2,2,5-trimethylhexane (12.92%), acetic acid (10.70%), 2-isothiocyanatobutane (9.71%), 4-chloro-1-methylpiperidine (6.33%) and d-glycero-d-ido-heptose (5.10%).

### 3.2. Antibacterial Activity

The inhibition zone diameters produced by the four extracts against the five fish pathogen isolates after 48 h of incubation are shown in Table A1, and those of the ten reference antibiotics in Table A2, representative plates are shown in Figure S1. No inhibition zones were observed around the solvent-control discs. Among the antibiotics, enrofloxacin, imipenem and levofloxacin were active against all five isolates (22.0–32.0 mm), whereas the *L. garvieae*, *Y. ruckeri* and *E. tarda* isolates showed no measurable zones with six to seven of the ten antibiotics tested under the present assay conditions, including oxytetracycline, sulfamethoxazole-trimethoprim and trimethoprim (Table A2). Of the 60 extract–dose-isolate combinations tested, 38 (63%) produced a measurable inhibition zone: 9 of 15 for the EC extract, 7 of 15 for EA, and 11 of 15 for each of MC and MA. The number of active combinations increased with disc loading (8 of 20 at 100 µg, 14 of 20 at 250 µg and 16 of 20 at 500 µg per disc) and differed markedly among isolates (*L. garvieae* 11 of 12, *V. anguillarum* and *Y. ruckeri* 10 of 12 each, *A. hydrophila* 6 of 12, *E. tarda* 1 of 12). For the four isolates with more than one active extract, two-way ANOVA on the complete 4 × 3 design indicated significant effects of extract, dose and their interaction (all *p* < 0.001), and the Kruskal–Wallis test across the 12 extract–dose combinations gave the same result (H = 33.3–34.9, *df* = 11, *p* < 0.001; Table A5); given the significant interactions, the pairwise Tukey comparisons reported in Table A1 were interpreted as simple effects, that is, among extracts within each dose and among doses within each extract. When the analysis was restricted to the active combinations, the same effects remained significant (extract and dose *p* < 0.001, interaction *p* <= 0.005; Table A3), but the relative standing of the extracts differed: against *A. hydrophila* the EC extract had the highest mean among the active cells (22.7 mm at its single active dose), whereas over the complete design the MC extract ranked first (18.0 versus 11.6 mm), and against *L. garvieae* the EA extract fell from the second to the fourth rank. Where inhibition occurred, zone diameters generally increased with dose: of the eight extract–isolate combinations with three active dose levels, seven showed significant positive dose–response slopes in the log-dose regressions (Table A4). The steepest slope was obtained for the MC extract against *A. hydrophila* (6.2 mm per doubling of dose, 95% CI 5.5–6.8). Among the six combinations with only two active dose levels, the largest descriptive difference was observed for the MC extract against *V. anguillarum* (9.0 mm between the 250 and 500 µg loadings); for these combinations only the pairwise Tukey comparison given in Table A1 is reported and no dose–response slope was estimated, because two dose levels do not allow the shape of the dose–response relationship to be assessed. In contrast, the MC extract against *Y. ruckeri* was active already at 100 µg per disc with no further significant increase across doses (slope 0.3 mm per doubling, *p =* 0.22; Table A4).

Against *A. hydrophila*, the MC extract was the most effective, producing inhibition zones of 10.7 ± 0.6, 18.3 ± 0.6 and 25.0 ± 1.0 mm at 100, 250 and 500 µg of dry extract per disc, respectively, whereas the EA extract produced no inhibition at any dose; at 500 µg, the MC extract zone was significantly larger than those of chloramphenicol and oxytetracycline (Welch’s *t*-test, Holm-corrected *p* < 0.01) and did not differ significantly from those of ampicillin, tetracycline, imipenem or streptomycin. Against *V. anguillarum*, the EA extract was the most active at all doses (17.7–23.7 mm), and the MC extract reached a comparable zone diameter (23.7 ± 0.6 mm) at 500 µg per disc; at this dose, both extracts produced significantly larger zones than tetracycline (18.0 ± 1.0 mm) and chloramphenicol (14.0 ± 1.0 mm) (Holm-corrected *p* < 0.05), did not differ significantly from ampicillin (16.0 ± 2.0 mm), and remained below the zones of enrofloxacin, imipenem and levofloxacin (26.0–28.0 mm). Against *Y. ruckeri*, the largest inhibition zone was obtained with the MA extract at 500 µg per disc (24.0 ± 1.0 mm), followed by the MC extract (20.7–21.3 mm), which was active even at the lowest dose tested; the MA extract zone did not differ significantly from that of ampicillin (23.0 ± 1.0 mm). Against *L. garvieae*, the MC and MA extracts produced the largest zones at 500 µg per disc (18.7 ± 0.6 mm); although these values remained below those of the three active antibiotics (22.0–31.7 mm), this isolate showed no zone with the remaining seven antibiotics of the panel, including ampicillin and tetracycline, to which the extracts were accordingly the only inhibitory agents besides enrofloxacin, imipenem and levofloxacin. The *E. tarda* isolate was the least susceptible to the extracts, being inhibited only by the EC extract at 500 µg per disc (12.3 ± 0.6 mm), whereas no inhibition zone was observed for the other extracts at any dose.

## 4. Discussion

In the present study, we investigated, to our knowledge for the first time, the antibacterial activity of *R. aculeatus* extracts against bacterial fish pathogens. All four extracts exhibited measurable, dose-dependent activity against at least one of the five pathogens tested, and four of the five pathogens (*A. hydrophila*, *V. anguillarum*, *Y. ruckeri* and *L. garvieae)* were inhibited by at least two extracts. These results suggest that the antibacterial activity of *R. aculeatus* previously reported against human clinical isolates may also extend to bacterial pathogens of farmed fish; however, as a single field isolate per species was tested, this conclusion requires confirmation with additional strains.

Our findings are consistent with previous studies on the antimicrobial potential of the genus *Ruscus*. Hadžifejzović et al. [23] reported that extracts and isolated compounds of *R. aculeatus* and *R. hypoglossum* exhibited antibacterial and especially antifungal activity, which in some cases exceeded that of standard drugs. Similarly, Edziri et al. [24] found that ethyl acetate and chloroform extracts of *R. hypophyllum* were active against multidrug-resistant bacteria, with MIC between 0.125 and 0.5 mg/mL, and Rodrigues et al. [18] demonstrated that hydroethanolic and aqueous extracts of *R. aculeatus* inhibited several multiresistant clinical strains. The activity observed in the present study against Gram-negative fish pathogens such as *A. hydrophila*, *V. anguillarum* and *Y. ruckeri*, as well as against the Gram-positive *L. garvieae*, therefore complements the existing evidence obtained with clinically relevant bacteria. The inhibition zones observed in this study were within the range reported for ethanolic plant extracts against the same fish pathogens in disc diffusion assays [10,11] and for other natural products such as propolis against *A. hydrophila* and *Y. ruckeri* [31], although differences in extract preparation and disc loading preclude direct quantitative comparison.

The differences in activity among the four extracts may be related to their chemical composition, which varied markedly with the solvent and the plant part used. The major constituents identified in this study included phytol, n-hexadecanoic acid, 9,12,15-octadecatrienoic acid methyl ester, 2-isothiocyanatobutane and 2,3-dihydro-3,5-dihydroxy-6-methyl-4H-pyran-4-one. Plant-derived secondary metabolites, including fatty acids, terpenoids and sulfur-containing compounds, are widely recognized as natural antimicrobial agents [5], and saponins, which constitute the characteristic bioactive fraction of *Ruscus* species [19,32], may also have contributed to the observed activity, although they are not amenable to detection by the GC–MS approach used. It should be noted, however, that saponins can also act as anti-nutritional factors, binding minerals and interfering with nutrient absorption [19]; this dual character would need to be considered if *R. aculeatus* preparations were to be developed as feed additives. Further studies employing complementary analytical techniques will be required to identify the compounds responsible for the antibacterial effects.

When compared with the reference antibiotics, the extracts generally produced smaller inhibition zones than the most active agents of the panel (enrofloxacin, imipenem and levofloxacin), which is expected considering that crude extracts were tested against purified compounds applied at standardized doses. Nevertheless, with all comparisons based on triplicate assays, the EA and MC extracts at 500 µg per disc produced significantly larger zones than tetracycline and chloramphenicol against *V. anguillarum*, and the MC extract zone against *A. hydrophila* exceeded those of chloramphenicol and oxytetracycline; moreover, the extracts inhibited the *L. garvieae* isolate, for which seven of the ten antibiotics tested, including ampicillin and tetracycline, produced no measurable zone. It should be noted, however, that disc diffusion comparisons between crude extracts and antibiotic discs are influenced by differences in diffusion behaviour, disc loading and formulation; these comparisons therefore describe relative performance under the assay conditions rather than equivalence of potency and require confirmation by the determination of MIC. The susceptibility patterns observed for the isolates themselves—particularly the absence of measurable zones for the *L. garvieae*, *Y. ruckeri* and *E. tarda* isolates with six or more of the antibiotics tested under the present assay conditions, underline the relevance of searching for alternative antimicrobial agents in aquaculture.

The use of natural products as antimicrobial agents has attracted increasing attention in food and aquaculture systems, both as alternatives to synthetic preservatives and in response to the spread of antibiotic resistance [5,33,34,35]. Formulation approaches such as combinations with biopolymers or nanoparticle carriers have been reported to enhance the efficacy of plant-derived antimicrobials and feed additives in fish [36,37,38,39,40,41,42].

Some limitations of the present study should be acknowledged. Only one field isolate per pathogen species was tested, and susceptibility to plant extracts may vary among strains of the same species; the present results should therefore not be generalized to the species level without confirmation. The antibacterial activity was evaluated only in vitro and at the level of inhibition zone diameters; MIC and minimum bactericidal concentrations (MBC) were not determined, and no quality-control reference strain was included. Because no zone could be measured for the 22 inactive extract–dose–isolate combinations, the inferential comparisons of zone diameters are conditional on activity; an analysis of the complete design, with these combinations at the detection floor, is reported in Table A5 and supported the effects described above. In addition, the gravimetric extraction yield of each solvent–plant part combination was not recorded, so the extraction efficiency of the four preparations cannot be compared; the antibacterial assays were nevertheless standardized on an equal dry-extract mass basis (100, 250 and 500 µg per disc). Because disc diffusion is influenced by the molecular size, polarity and agar diffusivity of the extract constituents, inhibition-zone diameters alone do not allow the intrinsic antimicrobial potency of the four extracts to be compared directly; the present results should therefore be regarded as a preliminary screening. Species assignments of the isolates were based on 16S rRNA gene sequencing, which has limited discriminatory power at the species level within some genera, notably *Aeromonas*, *Vibrio* and *Edwardsiella*; confirmation by multilocus or genome-based approaches would strengthen the identifications. In addition, the rhizome, which is regarded as the pharmacologically most important organ of *R. aculeatus*, was deliberately excluded to avoid destructive harvesting of natural populations; the antibacterial potential of rhizome extracts against fish pathogens therefore remains to be evaluated. Finally, cytotoxicity and in vivo efficacy remain to be assessed. Further studies should therefore include the determination of MIC and MBC values, cytotoxicity assays, in vivo challenge trials, and, if the extracts are to be administered as feed additives, an evaluation of their effects on the intestinal microbiota of fish.

## 5. Conclusions

In the present study, ethanol and methanol extracts obtained from the cladodes and the aerial parts of *R. aculeatus* exhibited in vitro antibacterial activity in this preliminary screening study against bacterial fish pathogens, with dose-dependent responses in most of the extract–pathogen combinations in which activity occurred. Because a single field isolate per pathogen species was tested, all comparisons among pathogens reflect the responses of the specific isolates examined and should not be generalized to the species level. The EC extract was the only extract that inhibited all five isolates, whereas the MC extract produced the largest inhibition zones against *A. hydrophila* and, together with the EA extract, against *V. anguillarum*; the *E. tarda* isolate was the least susceptible. To our knowledge, this is the first report of antibacterial activity of *R. aculeatus* extracts against bacterial fish pathogens. These preliminary in vitro findings suggest that *R. aculeatus*, a medicinal plant occurring naturally in Türkiye, deserves further evaluation as a potential source of plant-derived antimicrobial agents for aquaculture; however, the determination of MIC and MBC values, cytotoxicity tests, testing of additional isolates per species, and in vivo challenge trials will be required before any practical application can be considered.

## Figures and Tables

**Table 1:** Bacterial fish pathogen isolates used in this study, their host species of isolation and GenBank 16S rRNA gene accession numbers *.

Organism	Code	Description	Accession Numbers
*Aeromonas hydrophila*	SY-AH2	*Oreochromis niloticus*	MG844996
*Lactococcus garvieae*	SY-LG1	*Oncorhynchus mykiss*	KY118086
*Vibrio anguillarum*	SY-L24	*Dicentrarchus labrax*	KX388236
*Edwardsiella tarda*	SY-ED1	*Oreochromis niloticus*	KY126838
*Yersinia ruckeri*	E42	*Oncorhynchus mykiss*	KX388238

* Species designations follow the original deposition of these isolates [29]; identification was based on 16S rRNA gene sequencing.

**Table 2:** Phenotypic and biochemical characteristics of the five tested isolates.

Test/Characteristic	*A. hydrophila*	*L. garvieae*	*V. anguillarum*	*E. tarda*	*Y. ruckeri*
Gram reaction	−	+	−	−	−
Cell shape	Small rods	Cocci in pairs and short chains	Curved/short rods	Rods	Rods
Catalase	+	−	+	+	+
Oxidase	+	−	+	−	−
Glucose metabolism (O/F)	F	F	F	F	F
Indole	+	−	−	+	−
Urease	−	−	−	−	−
Lysine decarboxylase	+	−	−	+	+
Ornithine decarboxylase	−	−	−	+	+
Arginine dihydrolase	+	+	+	−	−
H2S	−	−	−	+	−
Mannitol	+	+	+	−	+
Sucrose	+	V	+	V	−
Growth without NaCl (0%)	+	+	−	+	+

+, positive; −, negative; V, variable (strain-dependent); F, fermentative. *V. anguillarum* appears in the source as *Listonella anguillarum*; for *Y. ruckeri* the manual recommends reading the reactions at 25 °C, because motility and citrate are negative at 37 °C. Reactions were determined in this study and interpreted according to Buller [28].

**Table 3:** Major chemical constituents (GC–MS; peak area ≥ 5%) of the EC extract of *R. aculeatus*. Identifications are tentative (NIST14 library matching); co-eluting alternatives are separated by a slash. Compounds are listed in decreasing order of relative peak area *.

Compound	RT (min)	Area (%)
Phytol	64.02	25.16
2,7-Octadiene-1,6-diol, 2,6-dimethyl/1,2,3,4-Butanetetrol, [S-(R,R)]-	56.27	16.52
1,2-Dimethylaziridine/Hexane, 2,2,4-trimethyl-	46.69	10.51
Neophytadiene/Undecanal	48.61	10.36

* Values are relative chromatographic abundances obtained by area normalization of the total ion chromatogram and do not represent absolute concentrations. The remaining 28 minor constituents (each < 5% of total peak area; cumulative 37.45%) are not shown. Major constituents listed account for 62.55% of the total peak area.

**Table 4:** Major chemical constituents (GC–MS; peak area ≥ 5%) of the EA extract of *R. aculeatus*. Identifications are tentative (NIST14 library matching); co-eluting alternatives are separated by a slash. Compounds are listed in decreasing order of relative peak area *.

Compound	RT (min)	Area (%)
n-Hexadecanoic acid	61.49	42.08
1,2,3,4-Butanetetrol, [S-(R,R)]-	56.39	16.05
2-Cyclohexen-1-one, 4-(3-hydroxybutyl)-3,5,5-trimethyl-	49.13	12.66
Phytol	64.01	8.77
1-Butanol, 3-methyl-	19.44	6.98

* Values are relative chromatographic abundances obtained by area normalization of the total ion chromatogram and do not represent absolute concentrations. The remaining 12 minor constituents (each < 5% of total peak area; cumulative 13.48%) are not shown. The major constituents listed account for 86.54% of the total peak area.

**Table 5:** Major chemical constituents (GC–MS; peak area ≥ 5%) of the MC extract of *R. aculeatus*. Identifications are tentative (NIST14 library matching); co-eluting alternatives are separated by a slash. Compounds are listed in decreasing order of relative peak area *.

Compound	RT (min)	Area (%)
Butane, 2,2-dimethyl/1,2-Dimethylaziridine/Oxalic acid, isobutyl pentyl ester	46.73	22.21
9,12,15-Octadecatrienoic acid, methyl ester, (Z,Z,Z)-	62.87	14.64
Phytol	64.02	7.86
Butane, 2-isothiocyanato-	52.41	7.65

* Values are relative chromatographic abundances obtained by area normalization of the total ion chromatogram and do not represent absolute concentrations. The remaining 29 minor constituents (each < 5% of total peak area; cumulative 47.61%) are not shown. The major constituents listed account for 52.36% of the total peak area.

**Table 6:** Major chemical constituents (GC–MS; peak area ≥ 5%) of the MA extract of *R. aculeatus*. Identifications are tentative (NIST14 library matching); co-eluting alternatives are separated by a slash. Compounds are listed in decreasing order of relative peak area *.

Compound	RT (min)	Area (%)
4H-Pyran-4-one, 2,3-dihydro-3,5-dihydroxy-6-methyl-	55.57	16.41
Hexane, 2,2,5-trimethyl-	46.70	12.92
Acetic acid	29.73	10.70
Butane, 2-isothiocyanato-	52.41	9.71
Piperidine, 4-chloro-1-methyl-	52.56	6.33
d-Glycero-d-ido-heptose	56.27	5.10

* Values are relative chromatographic abundances obtained by area normalization of the total ion chromatogram and do not represent absolute concentrations. The remaining 32 minor constituents (each < 5% of total peak area; cumulative 38.83%) are not shown. The major constituents listed account for 61.17% of the total peak area.

## Data Availability

The original contributions presented in this study are included in the article/Supplementary Material. Further inquiries can be directed to the corresponding author.

## Supplementary Materials

The following supporting information can be downloaded at: https://www.mdpi.com/article/10.3390/vetsci13090945/s1, Figure S1: Disc-diffusion assay plates of *Ruscus aculeatus* extracts against five bacterial fish pathogens.

## Abbreviations

The following abbreviations are used in this manuscript:

EC	Ethanol-extracted cladodes
EA	Ethanol-extracted aerial parts
MC	Methanol-extracted cladodes
MA	Methanol-extracted aerial parts
MBC	Minimum bactericidal concentrations
MIC	Minimum inhibitory concentrations
HSD	Honestly significant difference (Tukey’s test)
GC–MS	Gas chromatography–mass spectrometry
NIST	National Institute of Standards and Technology (NIST14 mass spectral library)
RT	Retention time
CI	Confidence interval
rRNA	Ribosomal RNA

## Appendix A

**Table A1:** Inhibition zone diameters (mm) produced by *R. aculeatus* extracts against five bacterial fish pathogen isolates after 48 h of incubation at 24 °C. Disc loadings of 100, 250 and 500 µg of dry extract per disc were applied as 10, 25 and 50 µL of a 10 mg/mL extract stock solution.

Pathogen	Dose (µg/disc)	EC	EA	MC	MA
*A. hydrophila*	100	–	–	10.7 ± 0.6 cA	–
	250	–	–	18.3 ± 0.6 bA	11.7 ± 0.6 bB
	500	22.7 ± 0.6 aB	–	25.0 ± 1.0 aA	15.7 ± 0.6 aC
*V. anguillarum*	100	–	17.7 ± 0.6 cA	–	11.0 ± 1.0 bB
	250	16.7 ± 0.6 aB	21.3 ± 0.6 bA	14.7 ± 0.6 bC	10.7 ± 0.6 bD
	500	17.3 ± 0.6 aB	23.7 ± 0.6 aA	23.7 ± 0.6 aA	14.3 ± 0.6 aC
*Y. ruckeri*	100	–	–	20.7 ± 0.6 aA	11.0 ± 1.0 cB
	250	8.7 ± 0.6 bC	17.3 ± 0.6 aB	20.7 ± 0.6 aA	21.0 ± 1.0 bA
	500	15.3 ± 0.6 aD	18.3 ± 0.6 aC	21.3 ± 0.6 aB	24.0 ± 1.0 aA
*L. garvieae*	100	9.7 ± 1.2 bC	–	14.3 ± 0.6 bA	12.0 ± 1.0 bB
	250	14.3 ± 1.5 aB	15.7 ± 0.6 aA	17.3 ± 0.6 aA	13.3 ± 0.6 bB
	500	15.7 ± 0.6 aB	16.3 ± 0.6 aB	18.7 ± 0.6 aA	18.7 ± 0.6 aA
*E. tarda*	100	–	–	–	–
	250	–	–	–	–
	500	12.3 ± 0.6	–	–	–

Values are expressed as mean ± standard deviation (*n* = 3). “–” denotes no inhibition zone in any of the three replicates, that is, a zone diameter equal to the 6 mm disc (the detection floor of the assay); these cells were included in the analysis at that floor and are not missing data. Within each pathogen, different lowercase letters (a–c) indicate significant differences among doses within the same extract and different uppercase letters (A–D) significant differences among extracts within the same dose (Tukey’s HSD test on the complete 4 × 3 design, *p* < 0.05). No letters are given for the *E. tarda* isolate, for which 11 of the 12 cells were at the detection floor. EC, ethanol cladodes extract; EA, ethanol aerial parts extract; MC, methanol cladodes extract; MA, methanol aerial parts extract.

**Table A2:** Inhibition zone diameters (mm) produced by ten reference antibiotic discs against the five bacterial fish pathogen isolates after 48 h of incubation at 24 °C.

Antibiotic (Disc Content)	*A. hydrophila*	*V. anguillarum*	*Y. ruckeri*	*L. garvieae*	*E. tarda*
Ampicillin (10 µg)	20.7 ± 0.6	16.0 ± 2.0	23.0 ± 1.0	–	22.0 ± 1.0
Imipenem (10 µg)	23.3 ± 0.6	26.0 ± 1.0	26.0 ± 1.0	22.0 ± 1.0	27.0 ± 1.0
Enrofloxacin (5 µg)	30.0 ± 1.0	26.0 ± 1.0	28.0 ± 2.0	29.0 ± 1.0	28.0 ± 1.0
Levofloxacin (5 µg)	26.0 ± 1.0	28.0 ± 1.0	32.0 ± 1.0	31.7 ± 1.5	29.3 ± 0.6
Tetracycline (30 µg)	21.7 ± 0.6	18.0 ± 1.0	–	–	–
Oxytetracycline (30 µg)	12.3 ± 0.6	–	–	–	–
Chloramphenicol (30 µg)	12.7 ± 0.6	14.0 ± 1.0	–	–	–
Streptomycin (10 µg)	23.0 ± 1.0	–	–	–	–
Sulfamethoxazole-trimethoprim (25 µg)	–	–	–	–	–
Trimethoprim (5 µg)	–	–	–	–	–

Values are expressed as mean ± standard deviation (*n* = 3); –: no inhibition zone. Disc contents are given in parentheses. Antibiotic susceptibility was determined by the Kirby–Bauer disc diffusion method on Mueller–Hinton agar under the same incubation conditions as the extract assays (48 h at 24 °C).

**Table A3:** Structure and results of the per-isolate two-way ANOVA models (extract × dose, type II sums of squares) fitted to the active extract–dose combinations. Doses of 100, 250 and 500 µg of dry extract per disc were applied as 10, 25 and 50 µL of a 10 mg/mL extract stock solution.

Isolate	Extracts Included (Active)	Doses (µg/disc)	Effect	*df*	*F*	*p*
*A. hydrophila*	EC, MC, MA	100, 250, 500	Extract	2, 12	228.2	<0.001
			Dose	2, 12	368.3	<0.001
			Extract × Dose	1, 12	12.0	0.005
*V. anguillarum*	EC, EA, MC, MA	100, 250, 500	Extract	3, 20	309.7	<0.001
			Dose	2, 20	165.2	<0.001
			Extract × Dose	4, 20	43.5	<0.001
*Y. ruckeri*	EC, EA, MC, MA	100, 250, 500	Extract	3, 20	252.8	<0.001
			Dose	2, 20	176.4	<0.001
			Extract × Dose	4, 20	74.5	<0.001
*L. garvieae*	EC, EA, MC, MA	100, 250, 500	Extract	3, 22	29.0	<0.001
			Dose	2, 22	105.6	<0.001
			Extract × Dose	5, 22	7.1	<0.001
*E. tarda*	EC only (single active cell)	500	Not analyzed	–	–	–

*df*, degrees of freedom (effect, residual); *F*, *F*-statistic; *p*-values < 0.05 were considered statistically significant. Models were fitted with type II sums of squares. Inactive combinations (no inhibition zone in any replicate) do not enter these models, so the extract and dose levels included differ among isolates, the non-estimable interaction contrasts arising from the resulting empty cells are dropped and the interaction degrees of freedom are reduced accordingly, and the designs are unbalanced; the effects reported here are therefore conditional on activity. The corresponding analysis of the complete 4 × 3 design of each isolate, with the inactive combinations coded at the assay detection floor, is given in Table A5. Shapiro–Wilk residual normality: *A. hydrophila p* = 0.040, *V. anguillarum p* = 0.007, *Y. ruckeri p* = 0.080, *L. garvieae p* = 0.118; Brown–Forsythe test *p* > 0.97 for all isolates. Kruskal–Wallis confirmation across the active extract–dose combinations treated as a single factor: H = 16.5 (*p* = 0.006), 28.1 (*p* < 0.001), 27.6 (*p* = 0.001) and 30.0 (*p* < 0.001) for *A. hydrophila*, *V. anguillarum*, *Y. ruckeri* and *L. garvieae*, respectively. For the *E. tarda* isolate, only a single extract–dose combination was active and no model was fitted. EC, ethanol cladodes extract; EA, ethanol aerial parts extract; MC, methanol cladodes extract; MA, methanol aerial parts extract. The primary analysis of the complete design is given in Table A5.

**Table A4:** Log2-dose regressions of inhibition zone diameter for extract–isolate combinations with three active dose levels, and descriptive dose differences for combinations with two active dose levels. Doses of 100, 250 and 500 µg of dry extract per disc were applied as 10, 25 and 50 µL of a 10 mg/mL extract stock solution.

Isolate	Extract	Active Dose Levels (µg/disc)	n	Slope (mm Per Dose Doubling)	95% CI	*p*	*R* ^2^	Mean Difference Between the Two Doses (mm)
*A. hydrophila*	MC	100, 250, 500	9	6.2	5.5–6.8	<0.001	0.99	–
	MA	250, 500	6	–	–	–	–	4.0
*L. garvieae*	EC	100, 250, 500	9	2.6	1.6–3.7	<0.001	0.84	–
	EA	250, 500	6	–	–	–	–	0.7
	MC	100, 250, 500	9	1.9	1.4–2.4	<0.001	0.92	–
	MA	100, 250, 500	9	2.8	1.5–4.0	0.001	0.80	–
*V. anguillarum*	EC	250, 500	6	–	–	–	–	0.7
	EA	100, 250, 500	9	2.6	2.1–3.0	<0.001	0.96	–
	MC	250, 500	6	–	–	–	–	9.0
	MA	100, 250, 500	9	1.3	0.2–2.5	0.027	0.53	–
*Y. ruckeri*	EC	250, 500	6	–	–	–	–	6.7
	EA	250, 500	6	–	–	–	–	1.0
	MC	100, 250, 500	9	0.3	−0.2–0.7	0.22	0.21	–
	MA	100, 250, 500	9	5.7	4.3–7.1	<0.001	0.93	–

Slopes represent the mean change in inhibition zone diameter (mm) per doubling of disc loading, estimated by ordinary least-squares regression of zone diameter on log2-transformed dose. n, number of observations (three replicates per active dose level); CI, confidence interval; *R*^2^, coefficient of determination. *p*, two-sided *p*-value for the test of the null hypothesis that the log2-dose slope equals zero (ordinary least-squares *t*-test); the level of significance was set at *p* < 0.05. For combinations with only two active dose levels, no regression was fitted; the mean difference in zone diameter between the two doses was reported instead. EC, ethanol cladodes extract; EA, ethanol aerial parts extract; MC, methanol cladodes extract; MA, methanol aerial parts extract. Combinations that were active at a single dose level only are not listed, namely the EC extract against the *E. tarda* isolate and the EC extract against the *A. hydrophila* isolate (both at 500 µg per disc).

**Table A5:** Per-isolate analysis of the complete 4 × 3 design of each isolate, with the inactive extract–dose combinations entered at the detection floor of the assay (zone diameter = 6 mm). Doses of 100, 250 and 500 µg of dry extract per disc were applied as 10, 25 and 50 µL of a 10 mg/mL extract stock solution.

Isolate	Effect	*df*	*F*	*p*	Kruskal–Wallis H (*df* = 11)	Kruskal–Wallis *p*
*A. hydrophila*	Extract	3, 24	979.3	<0.001	34.9	<0.001
	Dose	2, 24	1450.5	<0.001		
	Extract x Dose	6, 24	270.8	<0.001		
*V. anguillarum*	Extract	3, 24	416.3	<0.001	34.4	<0.001
	Dose	2, 24	835.8	<0.001		
	Extract x Dose	6, 24	116.6	<0.001		
*Y. ruckeri*	Extract	3, 24	481.9	<0.001	34	<0.001
	Dose	2, 24	549.3	<0.001		
	Extract x Dose	6, 24	79.1	<0.001		
*L. garvieae*	Extract	3, 24	49.6	<0.001	33.3	<0.001
	Dose	2, 24	239.5	<0.001		
	Extract x Dose	6, 24	19.6	<0.001		
*E. tarda*	Not analysed	-	-	-	-	-

Each cell of the complete design contained three replicates (*n* = 3); the designs are therefore balanced, and the type I, II and III sums of squares coincide. *df*, degrees of freedom (effect, residual). Inactive combinations (no inhibition zone in any replicate) were coded at the 6 mm disc diameter, that is, as measurements at the detection floor of the assay and not as missing values. Because these cells have zero within-cell variance, the residual mean square is reduced and the *F* values are to be read as confirmatory rather than as measures of effect magnitude; for this reason, the assumption-free Kruskal–Wallis test across the 12 extract–dose combinations of each isolate (H, *df* = 11) is reported alongside. For the *E. tarda* isolate, 11 of the 12 cells were at the detection floor and no model was fitted. Table A3 reports the same models fitted to the active combinations only and is therefore conditional on activity. EC, ethanol cladodes extract; EA, ethanol aerial parts extract; MC, methanol cladodes extract; MA, methanol aerial parts extract.
