# Supplementary material for: In Vitro Screening of Butcher’s Broom (Ruscus aculeatus L.) Cladodes and Aerial Parts Extracts Against Fish Pathogen Isolates
Source: Vet Sci. 2026 Sep 11;13(9):945. doi: 10.3390/vetsci13090945 (PMC13611905; doi:10.3390/vetsci13090945)
Supplement: Supplementary file 1 [file vetsci-13-00945-s001.zip › vetsci-4538426-supplementary.pdf]

Supplementary Figure S1. Disc-diffusion assay plates of *Ruscus aculeatus* extracts against five bacterial fish path-ogens

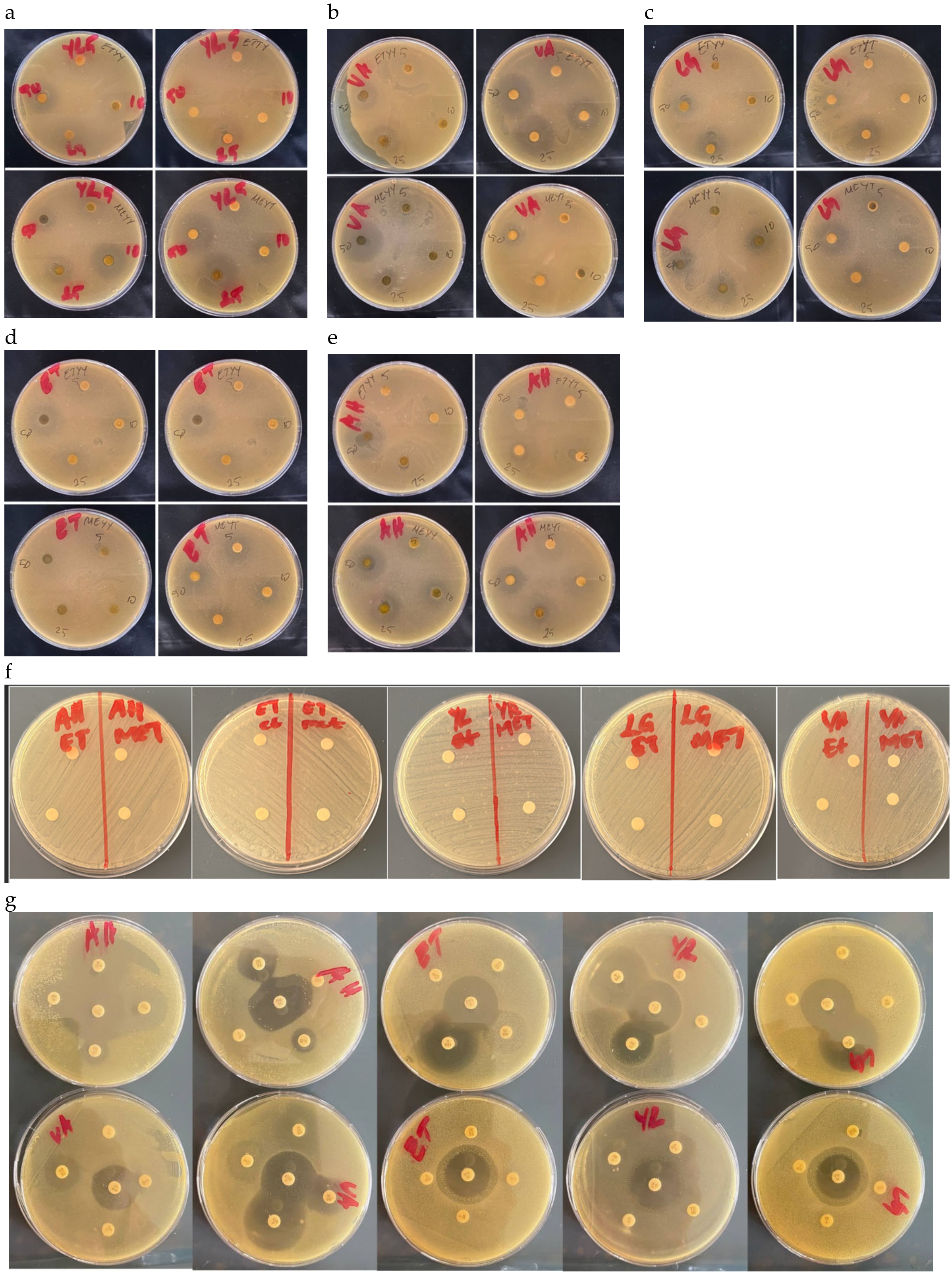

Figure S1. Disc-diffusion assay of butcher's broom (*Ruscus aculeatus* L.) extracts, of the extraction solvents and of the reference antibiotic panel against five bacterial fish pathogen isolates. Extract plates: (a) *Yersinia ruckeri* (E42); (b) *Vibrio anguillarum* (SY-L24); (c) *Lactococcus garvieae* (SY-LG1); (d) *Edwardsiella*

tarda (SY-ED1); (e) *Aeromonas hydrophila* (SY-AH2). In each of panels (a)–(e) the four plates correspond to the four extracts: EC (upper left), EA (upper right), MC (lower left) and MA (lower right). Every plate carries four extract discs; the numbers written on the agar beside the discs (5, 10, 25 and 50) are the applied volumes in μL of the 10 mg/mL extract stock solution, corresponding to 50, 100, 250 and 500 μg of dry extract per disc. The fourth, uppermost disc on each plate, marked "5", was loaded with 5 μL of the stock solution (50 μg of dry extract per disc); this dose produced no inhibition zone against any of the five isolates and was therefore not included in the manuscript. (f) Solvent-control plates. Each plate was inoculated with one isolate and divided into two halves by a marker line; one half received discs impregnated with 96% ethanol (marked "Et") and the other discs impregnated with ≥99.9% methanol (marked "MET"), two discs per half. No inhibition zone developed around any of these solvent-control discs. The plates shown are, from upper left, *A. hydrophila*, *E. tarda*, *Y. ruckeri*, *L. garvieae* and *V. anguillarum*. (g) Reference antibiotic panel. Two plates were used per isolate, each carrying five antibiotic discs, so that all ten antibiotics (ampicillin 10 μg, imipenem 10 μg, enrofloxacin 5 μg, levofloxacin 5 μg, tetracycline 30 μg, oxytetracycline 30 μg, chloramphenicol 30 μg, streptomycin 10 μg, sulfamethoxazole–trimethoprim 25 μg and trimethoprim 5 μg; Oxoid, Basingstoke, UK) were tested against every isolate under identical conditions.
